# A MEMS Pirani Vacuum Gauge Based on Porous Silicon †

**DOI:** 10.3390/mi16030296

**Published:** 2025-02-28

**Authors:** Yuzhe Lin, Zichao Zhang, Jifang Tao, Lianggong Wen

**Affiliations:** 1 School of Information Science and Engineering, Shandong University, Qingdao 266237, China; linyz@sdu.edu.cn (Y.L.); zc2020@mail.sdu.edu.cn (Z.Z.); 2 Shenzhen Research Institute of Shandong University, Shenzhen 518063, China; 3 Shenzhen Institute of Beihang University, Shenzhen 518057, China; wenlg@buaa.edu.cn

**Keywords:** Micro-Electro-Mechanical System, Pirani vacuum gauge, porous silicon, MEMS vacuum gauge

## Abstract

Vacuum gauges based on Micro-Electro-Mechanical System (MEMS) technology have the advantages of small size, high reliability, and low cost, so they are widely used in semiconductor, chemical, laboratory, and aerospace. In this paper, a high-reliability MEMS Pirani vacuum gauge based on a porous silicon platform is designed, fabricated, and characterized. The repeatability within 4~10^5^ Pa has been tested. The porous silicon acting as a support material achieved a porosity of 68% and a thermal conductivity of 3.5 W/(m·K), and the surface morphology of the porous silicon is smooth. The proposed MEMS Pirani vacuum gauge containing no suspended thin-film structures has good mechanical stability and is unaffected by mechanical shock and vibration in operation.

## 1. Introduction

The Pirani vacuum gauge measures pressure using the principle that the thermal conductivity of the gas varies as a function of pressure. Compared to conventional vacuum gauges, Pirani vacuum gauges based on advanced MEMS technology have been extensively studied due to their advantages of small size, high sensitivity, low power consumption, and low cost. MEMS Pirani vacuum gauges are commonly classified into microthermal bridge types [1], microthermal plate types [2], and multi-radiator microthermal trough types [3]. Microthermal plate type is mainly used to detect low-pressure range, while microthermal bridge type and micro hot slot type are mostly used to detect elevated pressure range. MEMS Pirani vacuum gauges are widely used in the semiconductor processing and manufacturing, aerospace, food processing, and chemical industries and also occupy a position in scientific research. Most current studies on air conduction vacuum gauge sensors employ levitated membrane structures [4,5,6,7,8,9,10,11,12,13,14,15,16,17,18,19,20], which are limited by the low strength of the membrane structure and the risk of membrane rupture and vacuum gauge failure under high temperatures and mechanical impact. The porous aluminum oxide structure has been studied [21]. However, the alumina (aluminum oxide) requires fabrication based on high-purity aluminum sheets, which involves a more complex process.

This paper studies the design of a MEMS vacuum gauge based on porous silicon as a thermal isolation layer, which greatly enhances the mechanical stability of the chip, making it able to withstand the strong convective shock generated at the moment of sudden changes in air pressure, and the risk of device failure caused by film rupture can be fundamentally solved.

## 2. Principle and Theoretical Analysis

**Figure 1:**
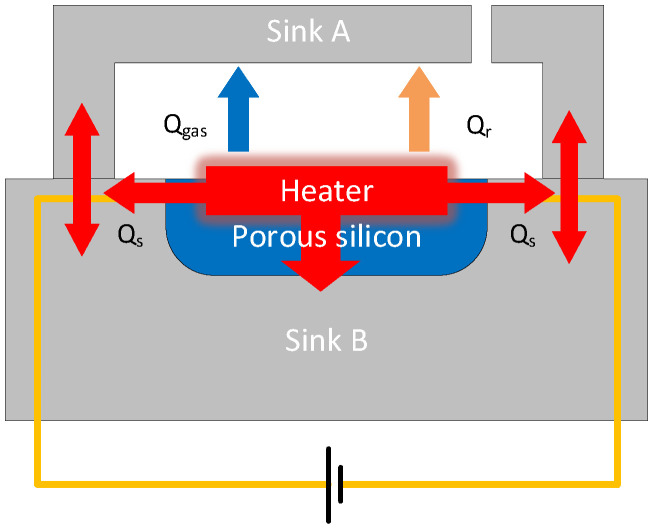
The principle of Pirani vacuum gauge.

Figure 1 shows a schematic illustration of the working principle of the MEMS Pirani vacuum gauge. The gas pressure (i.e., vacuum pressure) is measured based on the principle that heat transfer between two surfaces is proportional to the number of molecules transferring the heat if the mean free path of the gas molecules is larger than the distance between the surfaces. The core is that the change in the gas pressure will lead to a change in the thermal conductivity of the gas. In this study, the two faces are the face of Sink A and Heater.

The heat transfer processes of vacuum gauges during operation include Thermal convection, conduction, and radiation. The heating power of the heating electrode of the vacuum gauge is *Q_h_*. According to the law of conservation of energy, there is a functional relationship shown in (1):

(1) $$Q_{h}=Q_{gas}+Q_{s}+Q_{r}$$

$Q_{gas}$ represents the heat transferring between sink A and the heater through the gases, the blue arrow in Figure 1; $Q_{s}$ represents the heat conductivity through the solid part, the red arrow in Figure 1; $Q_{r}$ represents the thermal radiation, the orange arrow in Figure 1.

When the pressure changes and the $Q_{h}$ is kept unchanged, the temperature of the Heater should increase. The temperature change follows the pressure change. A numerical simulation model is built to investigate the characteristics of this sensor.

**Figure 2:**
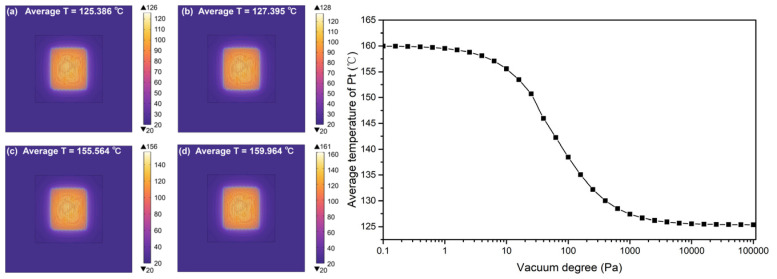
Simulation results of Pt temperature changing with the pressure. (**a**) P = 100,000 Pa (**b**) P = 1000 Pa (**c**) P = 10 Pa (**d**) P = 0.1 Pa.

Table 1 shows the design parameters of the sensor. With the simulation software COMSOL 6.0, the Pt temperature changing with the pressure is shown in Figure 2. The boundary condition is at room temperature (25 °C) and 32 V constant voltage. The highest temperature is 159.964 °C at 0.1 Pa, while the lowest temperature is 125.386 °C at 100,000 Pa. The temperature difference is almost 35 °C, which leads to the resistance change of 101 Ω with an original resistance of 750 Ω.

## 3. The Fabrication Process of Vacuum Gauge

**Figure 3:**
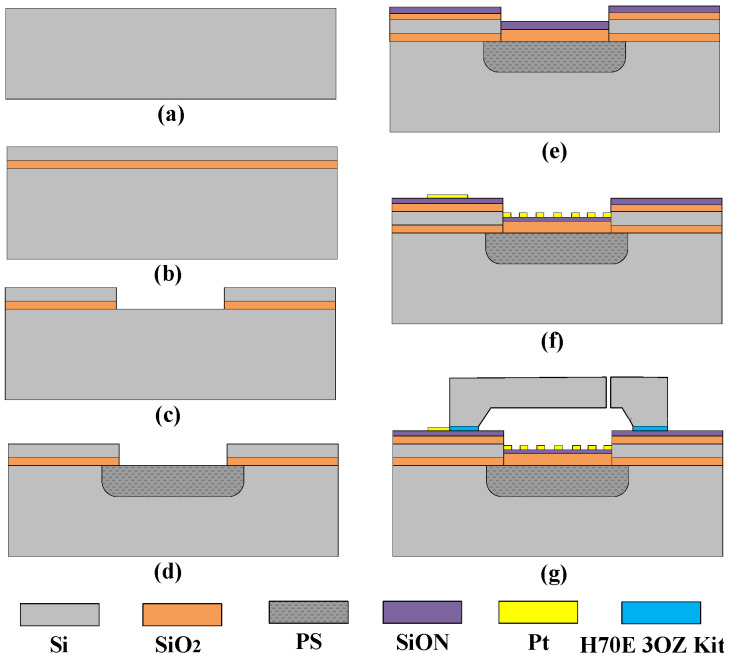
The fabrication process of MEMS vacuum gauge chip.

**Figure 4:**
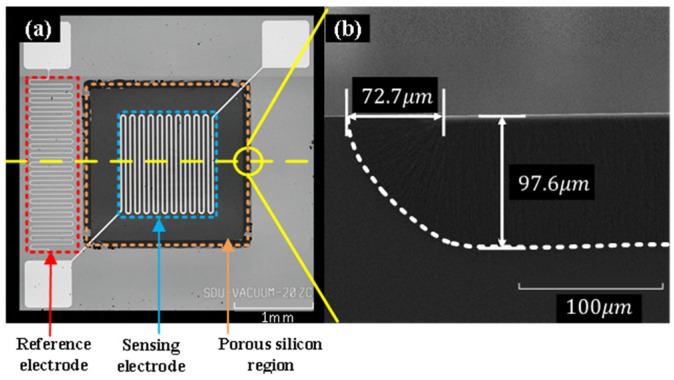
(**a**) The structure of the vacuum gauge chip. (**b**) Cross-section of porous silicon layer.

The process flow of the porous silicon vacuum gauge is shown in Figure 3 and described as follows [22]:

(a) Silicon wafer with P-type <100> Crystal orientation. (b) Sequential growth of silicon dioxide film and polysilicon film on the wafer. (c) ICP dry etching after the photolithography exposure process on the wafer, the film is etched to open a window for the subsequent etching of porous silicon. (d) Electron-chemical etching method is used to etch to form a regionalized porous silicon thermal insulation layer, and the etching result is as shown in Figure 4b; the depth of porous silicon is about 97.6 μm 0. (e) Silicon dioxide film and silicon oxynitride film are sequentially grown on the wafer surface, where porous silicon is etched to enhance the hydrophobicity of the chip surface. (f) Exposed by lithography after the process, a patterned metal electrode with a thickness of 200 nm and a width of 20 μm was formed on the wafer surface using electron beam evaporation technology. (g) Finally, a silicon cap with an air gap of 200 am was fabricated by bulk silicon etching technology and Bonded to a machined MEMS vacuum gauge with glue (H70E 3OZ Kit). Figure 4 is an SEM image of the chip after fabrication and a cross-section view of the chip.

For the porous silicon part, the porosity of the porous silicon can be calculated by the weighing method. The calculation is performed as follows:

(2) $$p=\frac{V_{P}}{V_{Si}}=\frac{(D/500)m_{1}-m_{2}}{(D/500)m_{1}-m_{3}}$$

where *V_P_* is the volume of the pores, *V_Si_* is the volume of the porous silicon layer, *m*_1_ is the mass of the silicon wafer before etching, *m*_2_ is the mass of the silicon wafer after forming the porous silicon and drying, *m*_3_ is the mass of the wafer after removing the porous silicon layer, and *D* is the thickness of the porous silicon layer. We have calculated that the porosity of our prepared porous silicon reaches 68.44%.

In addition, the thermal conductivity of porous silicon is also one of the key parameters for MEMS thermal devices. Raman spectroscopy was used to test the thermal conductivity of the prepared porous silicon sample. The basic principle of Raman spectroscopy test thermal conductivity is that the Raman spectral peak will shift to the left with the change in the temperature of the measured substance. The laser of Raman spectroscopy will increase the temperature of the porous silicon at the corresponding location. The thermal conductivity of porous silicon can be derived according to the correspondence between the temperature rise and the spectral peak. Perichon et al. derived a relationship between the local temperature rise of porous silicon and its thermal conductivity [23]:

(3) $$\lambda_{PS}=\frac{2P}{\pi a(T_{j}-T_{a})}$$

where *λ_PS_* is the thermal conductivity of porous silicon, *P* is the laser power causing temperature rise, *a* is the diameter of the laser beam spot, *T_j_* is the temperature rise caused by the laser, and *T_a_* is the substrate temperature.

**Figure 5:**
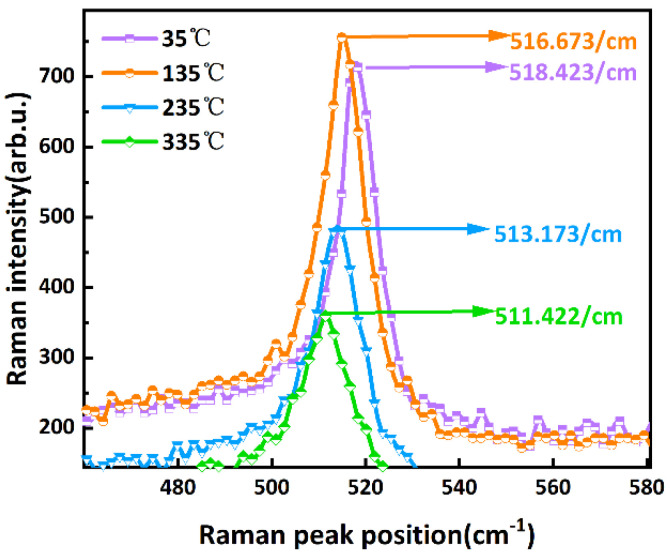
Raman spectra at different temperatures of porous silicon with 1 mW lasing power.

**Figure 6:**
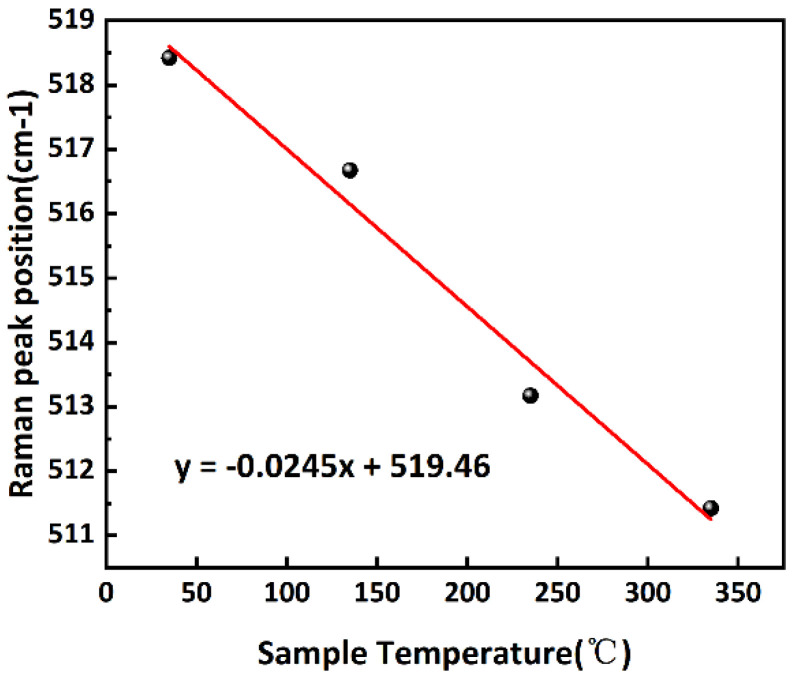
The relationship between temperature and Raman peaks of porous silicon samples.

The thermal conductivity test of porous silicon is performed as follows:

(1) Using a temperature control station to heat the porous silicon sample to 35 °C, 135 °C, 235 °C, and 335 °C in sequence.
(2) Using a lower-power (1 mW) laser to scan the Raman peak of the porous silicon sample at the above temperature in turn; the results are shown in Figure 5. From this, the correspondence between the Raman peak and the temperature of porous silicon is obtained, as shown in Figure 6.
(3) Scan the porous silicon sample with a high-power (5 mW) laser. Combined with the value of *T_j_* to calculate *λ_PS_*.

The final calculated thermal conductivity of porous silicon is 3.5 W/(m·K), which is 1/40 of the thermal conductivity of single crystal silicon, which can meet the thermal insulation requirements of MEMS Pirani vacuum gauge chips.

## 4. Measurement Results and Discussion

**Figure 7:**
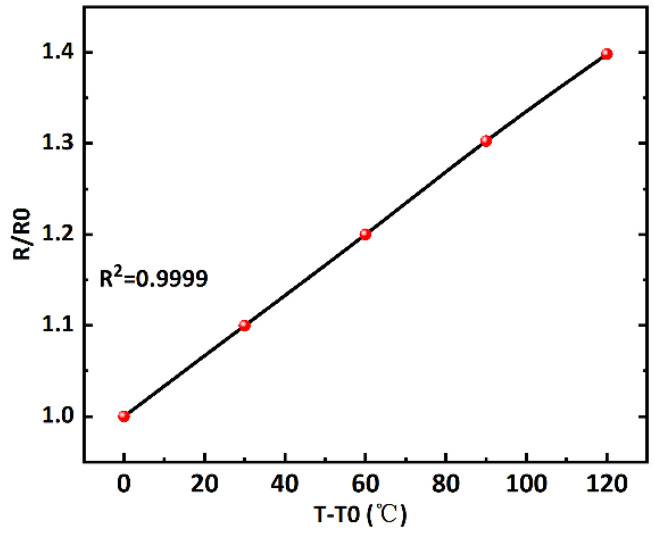
TCR of the platinum thin-film resistors.

First, the vacuum gauge chip is annealed, which stabilizes the resistance values of the metal electrodes and avoids affecting the test results. By examining the resistance values of the platinum resistors at different temperatures and plotting *R/R*_0_ versus temperature variation *T* − *T*_0_, we perform a linear fit on the points tested. The fitting results are shown in Figure 7, where R^2^ = 0.9999. The linearity is perfect, and the slope of the straight line, the temperature coefficient of resistance (TCR) of platinum, is 0.00333 K^−1^.

**Figure 8:**
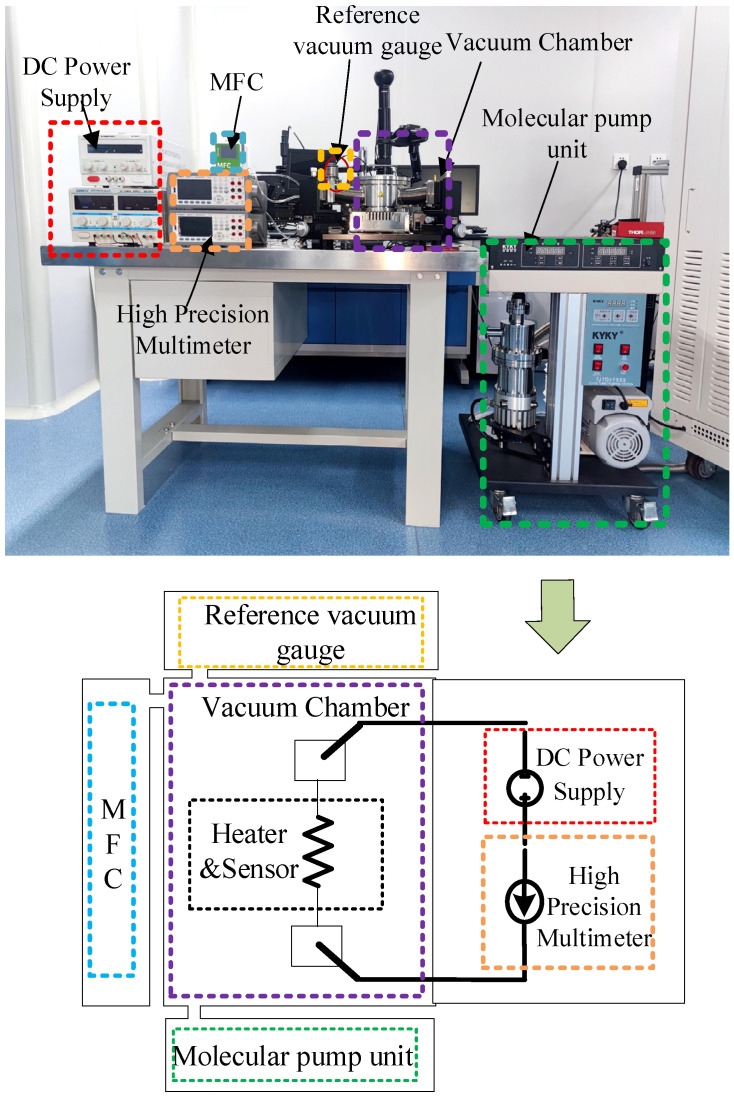
The schematic of the MEMS vacuum gauge test system.

The MEMS vacuum gauge test system consists of a vacuum chamber, a molecular pump unit, a high-precision current-voltmeter (34461A, Keysight, Santa Rosa, California, USA), a mass flow controller (MFC-T, Innovis, Qingdao, China), and a standard vacuum gauge, as shown in Figure 8. The output current of the MEMS vacuum gauge and the internal pressure of the cavity are measured by a high-precision multimeter and a standard vacuum gauge (TTR91N, Leybold, Cologne, Germany), respectively, where the output current value is obtained by connecting the sensitive resistor on the porous silicon in series with the constant voltage power supply.

The process of testing is as follows:

(1) Place the chip to be tested in the vacuum chamber and apply 32 V and 24 V voltages to it with a DC power supply, respectively;
(2) After using a molecular pump to pump the pressure in the vacuum chamber to the lowest pressure that can be pumped, the molecular pump is turned off, and the pressure value and output current value in the chamber are collected;
(3) The pressure in the cavity is adjusted by the gas flow controller, and when the pressure to be measured is stabilized, the pressure value and output current value at this time are recorded.

The collected data is processed to plot the response curve. In addition, the repeatability and stability of the MEMS vacuum gauge chip at normal temperature and pressure were tested.

**Figure 9:**
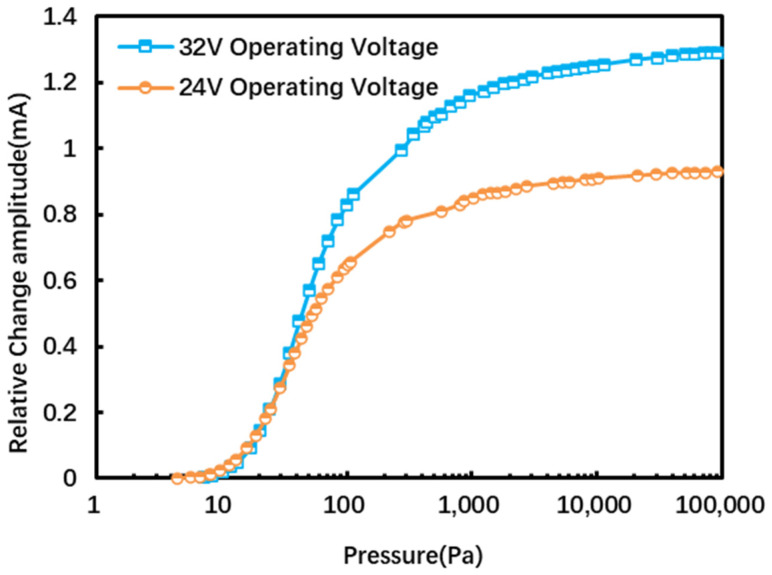
Vacuum response at different working voltages.

The vacuum response curves at two voltages, 32 V and 24 V, are tested and shown in Figure 9. It is shown that the vacuum gauge has a higher sensitivity at relatively high operating voltages. This is because the higher operating voltage will cause the platinum resistance to generate a higher temperature, and the external ambient temperature will be constant, so the temperature difference between the chip and the environment will be relatively higher, which is reflected in the chip’s sensitive resistance. The amplitude of the resistance shift is relatively larger; it can be seen from the resistance change value of Δ*R*, which is equal to *αR*_0_(*T_h_* − *T*_0_), that the sensitivity of the device with a large temperature difference will be higher (where *T_h_* is the temperature of the heating resistor, *T*_0_ is the ambient temperature, Δ*R* is the resistance value under *T_h_* and the difference between the resistance value under *T*_0_, *α* is the temperature coefficient of platinum resistance, and *R*_0_ is the resistance value at temperature *T*_0_). Therefore, we choose 32 V as the operating voltage of the MEMS vacuum gauge for better performance.

**Figure 10:**
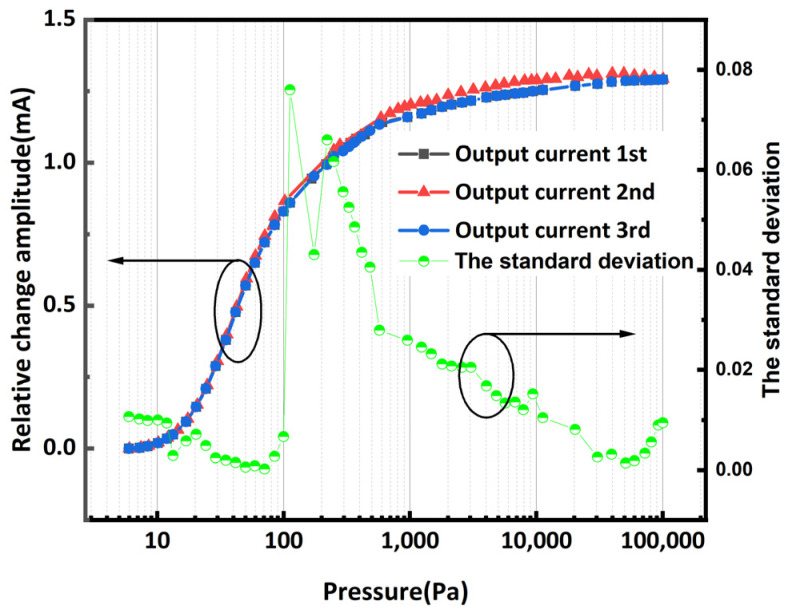
The testing results and standard deviation of three replicate tests.

The repeatability and stability of the chip are also critical performance metrics. Three repeatable experiments were performed on the same chip with the same test steps as above. The curves obtained from the three measurements are shown in Figure 10. The standard deviation is calculated over the entire measurement range. The standard deviation in the range of sensitivity is greater than the standard deviation in the range of low pressure and high pressure because the temperature in this area varies relatively greatly with air pressure, resulting in errors in the air pressure in the area that is difficult to control, but the standard deviation of the final data is within 0.08.

The proposed work based on a MEMS-compatible process porous silicon structure shows a wide pressure measuring range of 4–100,000 Pa. As shown in Table 2, compared with the membrane structure, the measuring range is similar, but the reliability is higher.

**Figure 11:**
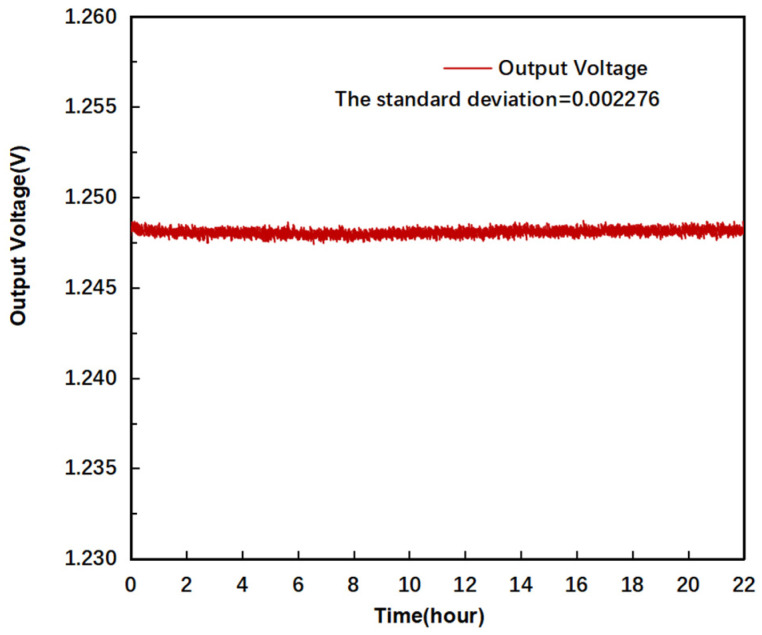
The stability test of vacuum gauge chip.

The stability of the chip was tested at a fixed ambient temperature (25 °C) and a fixed air pressure (1 atm). As shown in Figure 11, during the 22-hour test, the signal fluctuation was 0.0013 V, and the standard deviation was 0.002276. The noise was much smaller than the variation of the output amplitude, showing good stability and high detection accuracy under long-term operation.

## 5. Conclusions

In this paper, a MEMS vacuum gauge based on porous silicon is designed, fabricated, and characterized. The porous silicon fabrication process is optimized, which not only provides a stable supporting structure but also reduces the thermal conductivity to 3.5 W/(m∙K). This meets the requirements of high robustness and low thermal conductivity of MEMS vacuum gauge, e.g., the repeatability, stability, and a wide measurement range of 4 to 10^5^ Pa are tested. The proposed MEMS vacuum gauge addresses the poor mechanical properties and stability of the conventional membrane-type structures used in previous studies.

## Figures and Tables

**Table 1:** MEMS Pirani Vacuum Gauge Design parameters.

Parameters	Design Value
The thickness of porous silicon (μm)	100
Resisitance (Ω)	750
Area of Porous Silicon (μm)	1600 × 1600
Size of a sensor (mm)	3.2 × 3.2 × 0.5
Thickness of Air Gap (μm)	200

**Table 2:** Parameter comparison of MEMS Pirani vacuum gauge.

Researcher	Structure	Pressure Range (Pa)	Year
Kourosh Khosraviani [1]	Membrane	100–720,000	2009
Y Lv [4]	Membrane	0.001–1000	2025
X. Song [6]	Membrane	0.8–14,000	2024
M Garg [8]	Membrane	1–1,000,000	2024
G.-J. Jeon [21]	Porous AAO	0.013–101,324	2016
This work	Porous silicon	4–100,000	2025

## Data Availability

Data is contained within the article.
